# COVID-19 burden, author affiliation and women's well-being: A bibliometric analysis of COVID-19 related publications including focus on low- and middle-income countries

**DOI:** 10.1016/j.eclinm.2022.101606

**Published:** 2022-08-03

**Authors:** Lotus McDougal, Nabamallika Dehingia, Wendy Wei Cheung, Anvita Dixit, Anita Raj

**Affiliations:** Center on Gender Equity and Health, Department of Infectious Diseases and Global Public Health, School of Medicine, University of California San Diego. 9500 Gilman Dr, La Jolla, CA 92093, United States

**Keywords:** COVID-19, Gender, Bibliometric, LMIC, Authorship

## Abstract

**Background:**

Published literature documents tremendous gender inequities in the social, economic and health effects of the COVID-19 pandemic, but less evidence has come from low- and middle-income countries (LMICs) and even less from LMIC-based authors. We examine whether a) COVID-19 burden and b) LMIC-based authorship were associated with academic publications related to COVID-19 and women's well-being in LMICs.

**Methods:**

We reviewed academic articles on COVID-19 and women's well-being in LMICs published between February 2020 and May 2021 (*n=*1076 articles), using six electronic databases (PubMed, Web of Science, PsycInfo, EconLit, RePeC, NBER). Multilevel, mixed effects linear regressions assessed the relationships between each of our independent variables - a) COVID-19 burden (cases/100 population, deaths/100 population, deaths/cases) and b) author's country of primary affiliation, with publications related to COVID-19 and women's well-being, both overall and stratified by country income group.

**Findings:**

Eight-eight percent of articles had lead and/or senior authors affiliated with in-country institutions. Linear mixed effect models indicate that COVID-19 cases and case fatality ratios in a country were significantly and positively associated with the number of publications related to COVID-19 and women's well-being, though these relationships were significant only in upper-middle income group countries in stratified analyses. LMIC lead and senior authorship were also significantly and positively associated with our outcome, after adjusting for COVID-19 burden.

**Interpretation:**

While the majority of COVID-19 research examining women's well-being in LMICs in the first year and a half of the pandemic included country-affiliated author leadership, there were important gaps in representation. Findings highlight the importance of LMIC-based scholars to build local and gendered research in crises.

**Funding:**

Bill and Melinda Gates Foundation *(*INV-018007).

**Research in context**

**Evidence before this study**

We searched for articles indexed in PubMed (pubmed.gov), Web of Science (Clarivate), PsycINFO (ProQuest), EconLit (EBSCO), NBER (nper.org) and RePeC (repec.org)] for articles indexed by March 30, 2022 including “COVID-19” AND “gender” AND “bibliometric” AND (“LMIC” OR “low and middle income”). No papers were identified which looked at the relationship between COVID-19 burden and author affiliations with publications related to COVID-19 and women's well-being.

**Added value of this study**

Despite growing evidence that the COVID-19 pandemic has serious and detrimental gendered effects, there has been no comprehensive assessment to date of the ways in which publications related to COVID-19 and women's well-being vary across low and middle-income countries, what topics are being most commonly published on, and how COVID-19 burden and author affiliations relate to those publications. This bibliometric analysis fills these gaps by providing a statistical overview and assessment of publications related to COVID-19 and women's well-being including focus on low and middle-income countries over the first 16 months of the pandemic.

**Implications of all the available evidence**

While publications related to COVID-19 and women's well-being were generally reflective of COVID-19 burden in low and middle-income countries, these relationships were driven by upper-middle income countries, and were lacking in low-income and lower-middle income countries. Involvement of lead and/or senior authors affiliated with institutions in article focal countries was not associated with publications related to COVID-19 and women's well-being focused on economic impacts. There is a need for greater involvement of in-country authors on research examining a wider range of gendered COVID-19 impacts, as well as increased representation of diverse topics and publications related to COVID-19 and women's well-being focused on lower income countries.

Alt-text: Unlabelled box

## Introduction

As of early March 2022, the COVID-19 pandemic has killed more 5.9 million people worldwide, and infected more than 437 million.^1^ The social, economic and health effects of this pandemic are profound, far-reaching, and highly gendered in nature.^2^, ^3^, ^4^, ^5^, ^6^, ^7^, ^8^, ^9^, ^10^ COVID-19 infection rates and mobility reductions are associated with increased depression and anxiety, with more pronounced effects among women.^11^ Mobility restrictions have also placed women at more pronounced risk for gender-based violence, particularly from intimate partners, as well as inhibited access to key sexual, reproductive, and maternal health services.^12^, ^13^, ^14^ These access barriers are compounded by severe COVID-19-related contraceptive supply chain disruptions, restricting women's access to family planning care and essential contraceptive commodities.^7^^,^^13^ Women have been more impacted by job and wage loss due to their overrepresentation in heavily impacted industries such as tourism and hospitality, and face heavy burdens of unpaid care and childcare.^6^^,^^8^^,^^15^

These impacts are exacerbated in low- and middle-income countries (LMIC), where higher levels of existing gender inequalities, power imbalances, and regressive gender norms compound pandemic challenges.^16^^,^^17^ While LMICs bear a greater burden of gendered COVID-19 impacts, prior studies have indicated relatively lower representation of these regions in health-science related publications.^18^ The misalignment between disease burden and health research efforts is well-documented, with a disproportionate focus on high-income countries and their health needs and disease burdens.^19^, ^20^, ^21^ Scientific publication disparities also exist within LMICs, with upper-middle income countries generally more represented than low-income countries despite substantial disease burdens and adverse health outcomes in these less resources settings.^19^ Disparities in research efforts may be driven by a multitude of factors including, but not limited to, capacity of healthcare systems and National Statistical Offices, and environmental and economic conditions. Research productivity may also be influenced by support from development assistance funding, which has increased during the COVID-19 pandemic.^22^ Identifying the ways that these research publication disparities may have been impacted by the COVID-19 pandemic is an important opportunity to identify potential imbalances in research priorities and efforts, and to advocate for shifts, if necessary. While there have been many bibliometric studies of the influence of the COVID-19 pandemic on health topic-specific research trends,^23^, ^24^, ^25^, ^26^ no studies to date have focused on shifts in women's well-being related publications during the pandemic. A 2021 review of the literature on COVID-19 found very low representation of LMIC-focussed and non-medical research, or research on social and economic aspects of the pandemic.^27^ Considering scientific publications as a marker of research efforts, our study examines trends in research efforts for gendered impacts of the pandemic in LMICs, in the context of varying COVID-19 burden across countries.

Authorship is another important marker of representation in research efforts. Multiple empirical studies and opinion pieces by experts have pointed to the low representation of researchers from LMICs in research concerned with these regions.^18^^,^^28^, ^29^, ^30^ While a recent analysis of over 700,000 health science-related publications that focused on LMICs found an increase LMIC-affiliated authorship from 2000 to 2017, this change was driven mainly by increase in authorship from upper-middle income countries.^18^ The inclusion of local experts and diversity of scientists is a central component of efforts to decolonialize global health,^31^ and critical to making science more culturally sensitive, pragmatic, and actionable, and to be able to move away from a ‘foreign gaze’.^32^ Particularly in the context of gendered impacts of the pandemic, which have manifested in different forms across regions, it is important that local voices are highlighted and heard within the scientific community for effective policy action.

What has been learned about the gendered impacts of the COVID-19 pandemic is in large part derived from scientific publications.^33^ To date, however, the volume and characteristics of publications related to COVID-19 and women's well-being has not been comprehensively assessed. Published research is often not disaggregated by sex, may not include women participants, and may not adequately consider the ways that gender affects the mechanisms in question.^34^^,^^35^ In the absence of this information, there are barriers in understanding the gendered ways that COVID-19 affects different people in different circumstances. The objective of this paper is to address this gap by examining trends in academic publications related to COVID-19 and women's well-being in LMICs as a body of work in itself, and by examining specific topics within these women's well-being related publications. We use data generated from a large literature review to conduct bibliometric analyses assessing associations between our independent variables- a) COVID-19 burden and b) affiliations of authors in leadership roles, and our outcome, publications related to COVID-19 and women's well-being in LMIC contexts. To the best of our knowledge, this is the first bibliometric analysis of this kind.

## Methods

### Data

We conducted a recurring literature review of academic articles related to COVID-19 and women's well-being in LMIC. Six electronic bibliographic databases [PubMed (pubmed.gov), Web of Science (Clarivate), PsycINFO (ProQuest), EconLit (EBSCO), NBER (nper.org) and RePeC (repec.org)] were searched for peer-reviewed literature and working papers. The reviews were conducted every 1-2 weeks between June 2020 and May 2021, thus covering all relevant articles on COVID-19 and women's well-being indexed in the selected databases through 28^th^ May 2021. Our search criteria were developed by gender research experts, and included terms related to five broad thematic areas related to COVID-19 and women's well-being: a) Women and girls’ health, b) Gendered social impacts (including norms), c) Gendered economic impacts, d) Women's collectives, and e) Women's leadership (search terms in Appendix Table 1). Identified studies were then screened by a study researcher for the following eligibility criteria: inclusion of qualitative or quantitative data, findings or analysis focused on a LMIC, including any empirical analyses, and inclusion of any finding on the gendered aspects of social, economic and health impacts of the pandemic and spread containment responses in LMIC contexts. We excluded studies which had no empirical analysis (e.g. opinion or commentary pieces with no data analysis), were not in English, did not have full-text available, did not include focus on at least one LMIC, were missing basic study information (e.g. publication date, details on country/countries of focus, information on methods/analyses used), or were clearly off-topic. The study protocol was registered with Figshare (https://doi.org/10.6084/m9.figshare.12830513.v3).^36^ Study researchers (ND, WWC, AD) conducted reviews of included databases every 1-2 weeks throughout the study period. Search results were screened for eligibility and subsequently extracted. Study researchers met with study supervisors (LM, AR) every 2 weeks to review and validate all extracted data, as well as to resolve any queries.

**Table 1:** Descriptive statistics on publications related to COVID-19 and women's well-being with focus on low and middle income countries included in this review.

	Total	Focal area^1^				
		Women and girls’ health	Gendered social outcomes	Gendered economic impacts	Women's leadership	Women's collectives
	N (%)	N (%)	N (%)	N (%)	N (%)	N (%)
Number of publications	1076 (100 %)	975 (90·6%)	129 (12·0%)	57 (5·3%)	7 (0·7%)	1 (0·0%)
Lead or senior author affiliated with focal country						
Neither	134 (12·4%)	104 (10·7%)	29 (22·5%)	28 (49·1%)	4 (57·1%)	1 (100·0%)
Lead only	67 (6·2%)	63 (6·5%)	8 (6·2%)	1 (1·8%)	0 (0·0%)	0 (0·0%)
Senior only	41 (3·8%)	35 (3·6%)	9 (7·0%)	2 (3·5%)	1 (14·3%)	0 (0·0%)
Lead and senior/Lead in single author papers	834 (77·5%)	773 (79·3%)	83 (64·3%)	26 (45·6%)	2 (28·6%)	0 (0·0%)

1 Topics were not exclusive; some studies had more than one women's well-being related focal area.

**Figure 1:**
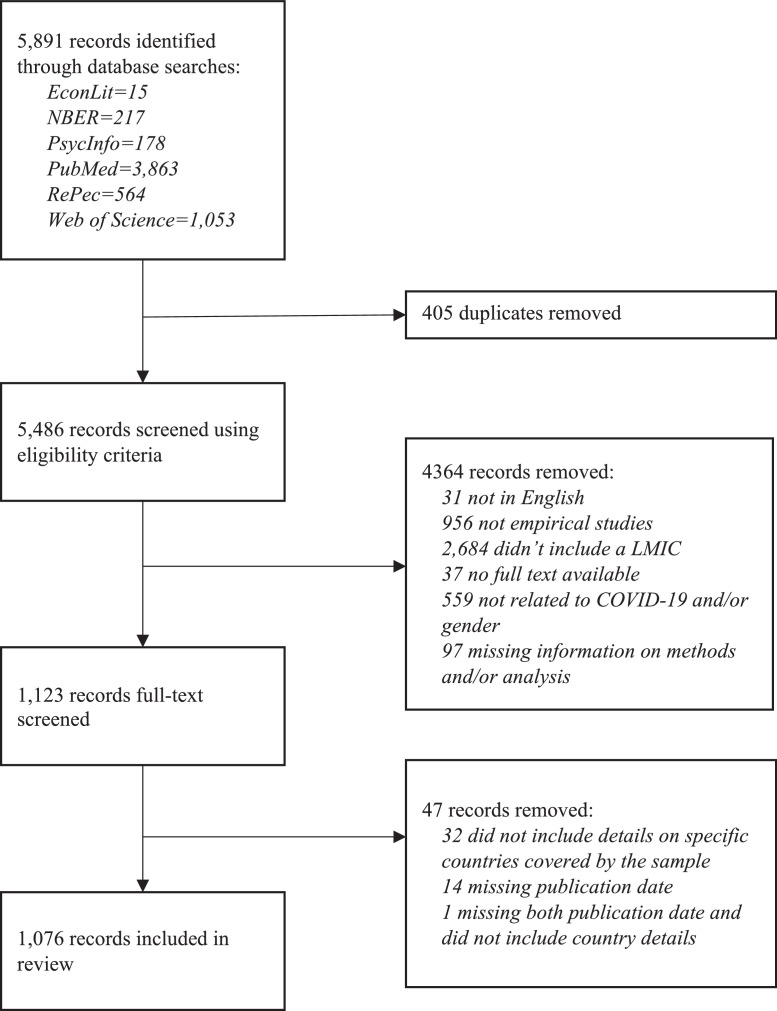
Study flow diagram.

Over 5800 articles were identified using our search criteria during the review period. Of these, 1123 studies were found to satisfy eligibility criteria (Figure 1). Data on date of publication, countries of focus for these eligible studies, and country of institutional affiliation of lead and senior authors were extracted and used for the current analysis. Thirty-three studies did not note their countries of focus, and fifteen studies did not provide date of publication (one study did not include both countries of focus and date of publication). These studies were excluded from the analysis. Data from a total of 1076 studies were thus included in the current analysis.

Four country-level variables were also included in this analysis. Country level data on COVID-19 cases and deaths were extracted from the WHO database.^1^ Countries were classified by income group (upper-middle income/ lower-middle income/ lower-income) using the World Bank income groupings.^37^ Gender Inequality Index (GII) 2019 rankings were obtained from the United Nations Development Program,^38^ and total development assistance received by countries from donors in 2019 was obtained from the Organization for Economic Co-operation and Development (OECD) database.^39^

### Measures

From our literature review, we calculated the number of academic publications on COVID-19 and women's well-being focusing on each LMIC for each of the 16 months of observation. These publications were further categorized by topical area of focus: women and girls’ health (inclusive of but not limited to COVID-19 infection), gendered social outcomes, gendered economic impacts, women's leadership, and women's collectives. Topical focal areas were not exclusive, thus articles could be noted as having two or more focal areas. We also reviewed the country of institutional affiliation for the lead and senior authors of each paper, and identified whether that country was the focal country of the article in question (or one of the focal countries, for articles which included more than one focal country). Authorship affiliations in the article focal country were categorized as neither lead nor senior author's primary institutional affiliation was in the focal country, lead only, senior only, or both lead and senior. Articles with a single author whose primary affiliation was in the focal country were categorized as both lead and senior authors being affiliated in the focal country.

Study quality and risk of bias are important tools in understanding the quality and composition of research included in scientific reviews. While we considered an assessment of study quality and risk of bias based on existing guidance,^40^ the studies encompassed in this recurring review were of such a diverse range of methodologies and disciplines that reviewing their quality in a way that was both meaningful and comparable across manuscripts presented a substantial challenge in terms of concept and application (an issue which has been noted elsewhere^41^). We thus opted not to analyze these assessments in this manuscript, instead focusing on a purely bibliometric lens; this is not meant to undermine the importance of study quality and risk of bias, which are essential components of many review structures.

We used three variables to measure COVID-19 burden in a country: number of COVID-19 cases per 100 population, number of COVID-19 deaths per 100 population, and COVID-19 case fatality ratio. The case fatality ratio was defined as the number of COVID-19 deaths per 100 COVID-19 cases in each country. These three variables were estimated for the overall review period, as well as monthly (June 2020 – May 2021).

Information related to income groups for the LMICs was based on the World Bank classification for the financial year 2021-22, which categorizes LMICs as upper-middle income, lower-middle income, and low-income. Venezuela was unclassified in 2021-22 by the World Bank, hence its categorization for 2019 was used in this analysis.

The GII is an aggregate measure of gender inequality calculated at the country level by the United Nations Development Program. The GII measures gender inequalities across three aspects of human development: reproductive health, measured by maternal mortality ratio and adolescent birth rates; empowerment, measured by proportion of parliamentary seats occupied by females and proportion of adult females and males aged 25 years and older with at least some secondary education; and economic status, expressed as labor market participation and measured by labor force participation rate of female and male populations aged 15 years and older. We used the GII for 2019 for each LMIC, with values ranging from 0 (gender equality) to 1 (gender inequality).

Total development assistance captured the total amount of development assistance funds received by each LMIC in 2019, from private donors, official development assistance, and other official flows. This measure is designed to represent the volume of external funds aimed at promoting growth in economic, health and social systems, as a proxy for support to in-country research infrastructures.^42^

### Statistical analysis

This bibliometric analysis summarizes data from the 1,076 articles eligible for review, providing cross-sectional, article-level descriptive frequencies of author primary institutional affiliations with the number of publications overall, and across assessed focal areas. Country-level descriptive analyses summarize focal topics, author affiliations, COVID-19 burden (cases, deaths and case fatality ratios), GII, and total development assistance for the 137 LMICs included in this sample overall and stratified by income group.

Multilevel, mixed effects linear regression models were used to assess the relationship between our independent variables - COVID-19 burden and authorship affiliations in a given country, and our outcome variable- the number of publications related to COVID-19 and women's well-being focusing on that same country. These models assessed these relationships by country using a longitudinal panel data structure to implement within-between models^43^^,^^44^ including time-varying effects (COVID-19 burden), time-invariant effects (country COVID-19 burden means, authorship affiliation means, income group, GII, and official development assistance) and random effects (country), while accounting for repeated (monthly) measures in each country over the study period. COVID-19 burden was modelled with a six-month lag to account for the delays between the effects of a given level of COVID-19 burden and publishing a scientific article about those effects. While scientific publications often take longer than six months to develop and publish, we used this shorter window given the accelerated peer review processes adopted by many scientific journals for COVID-19-related articles.^45^^,^^46^ The number of publications related to COVID-19 and women's well-being, COVID-19 burden and official development assistance were all right-skewed and are thus presented as natural logs in all regression models.

To fully understand the effect of country income group on the relationship between COVID-19 burden and publications related to COVID-19 and women's well-being, an additional exploratory analysis ran country income group-stratified iterations of the within-between models for each three COVID-19 burden predictors, again modeled using a six-month lag between COVID-19 burden.

A secondary analysis examined the relationship between our independent variables and the following outcomes- the number of women's well-being related publications focusing on that same country in three primary focal areas: women and girls’ health, gendered social outcomes, and gendered economic impacts. Models adjusted for all factors included in the main regression models.

All regression models were also tested with no lag between COVID-19 burden and publication date, but model fits were worse than those with the six-month lag (data not shown). We also modeled three- and nine-month lags for comparative purposes, and results did not differ meaningfully from the six-month lag presented below (data not shown).

All analyses were conducted in R, version 4.0.4.

### Role of the funding source

The funder had no role in manuscript writing, analysis, interpretation or submission. Authors were not precluded from accessing data in the study, and accept responsibility to submit for publication. All authors had access to the raw data, and all authors approved submission of the final manuscript for publication.

## Results

The 1076 women's well-being related COVID-19 articles reviewed and included in this analysis focus on 136 different LMICs. Three-quarters of reviewed articles (78%) were written by both first and senior authors with primary affiliations in the country (or one of the countries) of focus of that article; one in eight articles (78%) were neither lead- nor senior-authored by an individual with a primary affiliation matching that article's focal country. The majority of reviewed articles (91%) focused on women and girls’ health, with fewer focusing on gendered social outcomes (12%) and gendered economic impacts (5%) (Table 1). The most common sub-topics within women and girls’ health were mental health (46%) and maternal health (27%). Fewer than one percent of articles focused on women's leadership (*n=*7) or women's collectives (*n=*1); these two focal areas were thus excluded from regression-based analyses.

In-country authorship for both lead and senior authors was most common among articles focusing on women and girls’ health (79%), and least common among articles focusing on gendered economic impacts (46%) and women's leadership (29%). Nearly half of articles focusing on gendered economic impacts (49%) were authored by neither in-country affiliated lead nor senior authors. The single article focusing on women's collectives was authored by neither a lead nor senior author with in-country affiliation. In-country affiliation for lead and senior authors was much more common among papers focusing on a single focal country (lead author only 96%, senior author only 88%, lead and senior author 92%); affiliation of neither lead nor senior author with the focal country was more evenly distributed (60% single country articles, 40% multi-country articles).

Reviewed articles represented 2,175 country-articles (some articles focused on more than one LMIC). Women's well-being related COVID-19 articles in this review include all LMICs with the exception of the Democratic People's Republic of Korea (see Appendix Table 2). An average of 16 articles were indexed per country during the study review period, though this varied substantially by country income group (Table 2). Over the study period, an average of 21 articles/country were indexed with focus on upper-middle income countries, 15 articles per country with focus on lower-middle income countries, and eight articles per country with focus on low-income countries. More articles focused on women and girls’ health and gendered social outcomes in upper-middle income countries (an average of 18/country and 2/country, respectively), while more articles focused on gendered economic impacts in lower-middle income countries (an average of 1·2/country). In terms of authorship, more in-country affiliated lead authors published papers focused on lower-middle income countries (an average of 0·6/country), while in-country senior authorship, lead and senior authorship, and neither lead nor senior authorship focused on upper-middle income countries (an average of 0·4/country, 9·2/country and 10·8/country, respectively).

**Table 2:** Descriptive statistics on low- and middle-income countries include in this sample (*n=*136).

	Total sample	Country income group		
		Low	Lower-middle	Upper-middle
Publications related to COVID-19 and women's well-being (mean)	15·88	8·07	14·65	20·93
Women's well-being related focus^1^				
Women and girls’ health	13·47	5·93	12·22	18·42
Gendered social outcomes	1·96	1·33	1·98	2·24
Gendered economic impacts	0·95	0·63	1·18	0·87
Women's leadership	1·18	1·07	1·16	1·27
Women's collectives	0·01	0·00	0·02	0·00
Lead or senior author affiliated with focal country				
Neither	9·25	6·41	9·12	10·76
Lead only	0·53	0·30	0·60	0·56
Senior only	0·31	0·15	0·27	0·44
Lead and senior/ lead in single-author papers	5·79	1·22	4·65	9·16
ln COVID-19 cases per 100 population (mean)	0·78	0·11	0·54	1·34
ln COVID-19 deaths per 100 population (mean)	0·04	0·003	0·02	0·08
ln COVID-19 case fatality ratio (mean)	1·02	1·20	0·94	1·00
Gender Inequality Index (mean)	0·43	0·60	0·47	0·32
ln development assistance (USD, millions) (mean)	7·70	7·89	7·64	7·65

1 Topics were not exclusive; some studies had more than one women's well-being related focal area.

**Figure 2:**
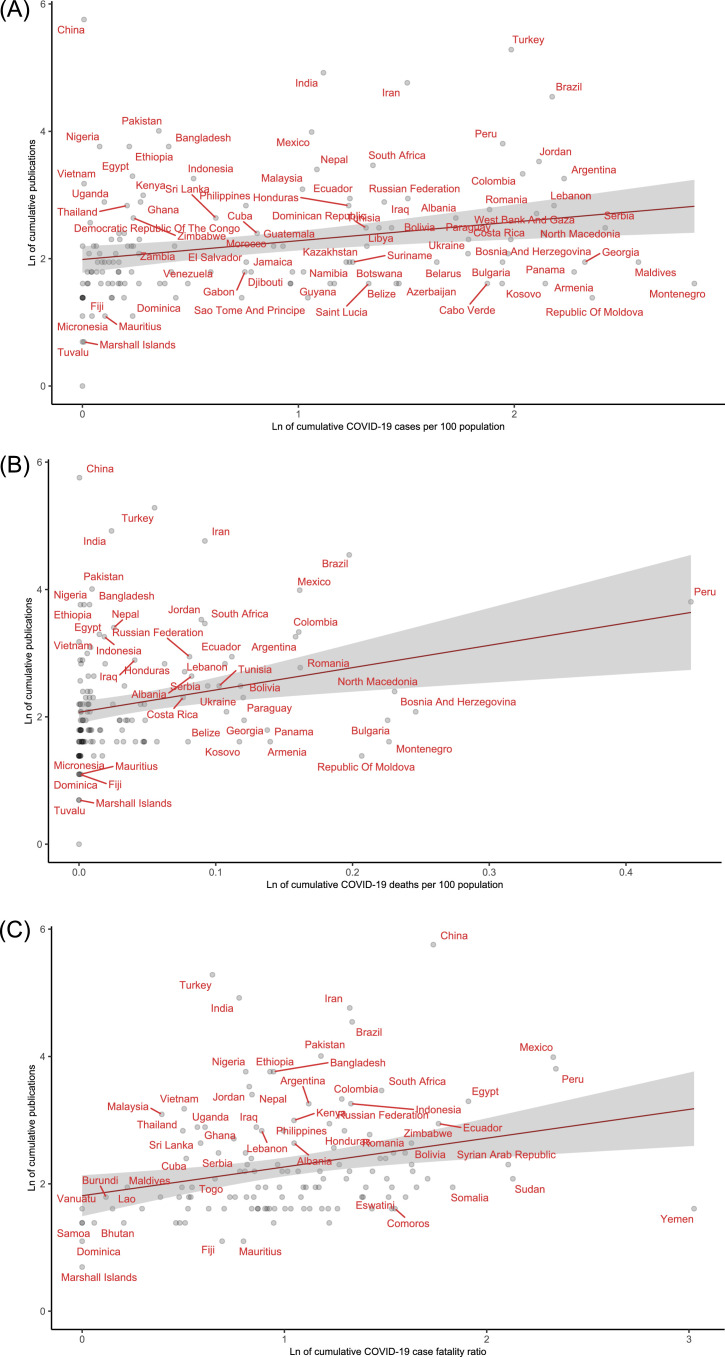
Scatterplots of the natural log of cumulative of publications related to COVID-19 and women's well-being with focus on LMICs and the natural log of cumulative COVID-19 cases per 100 individuals (A), deaths per 100 population (B), and case fatality ratios (C) over the study period. Note: *Lines are linear best-fit lines with shaded 95% confidence intervals.*

There was substantial variation in the relationship between COVID-19 burden and being a country of focus in publications related to COVID-19 and women's well-being (Figure 2). Generally, as COVID-19 cases, deaths and deaths/cases increased, publications related to COVID-19 and women's well-being also increased, though there were many outliers including China, Turkey, India and Brazil. These relationships were largely consistent across country income groups, with the exception of case fatality ratios in low-income countries (Appendix Figure 1).

**Figure 3:**
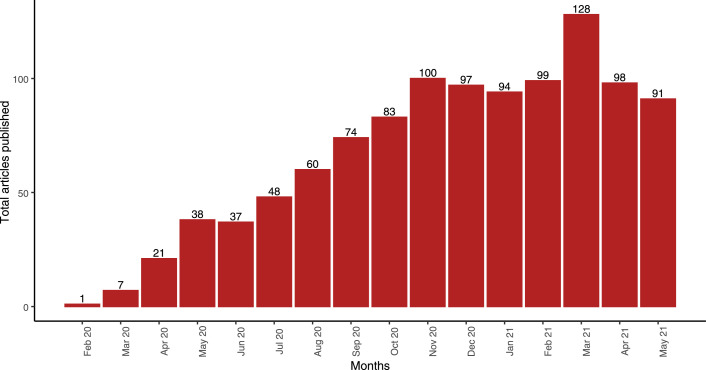
Monthly total publications related to COVID-19 and women's well-being with focus on any LMIC, between February 2020 and May 2021. Note: The figure indicates number of eligible articles identified until the last review date, i.e., 28^th^ May 2021. Due to the lag between publication and indexing in journal databases, numbers for April 2021 and May 2021 may be undercounting actual number of publications in those months.

**Figure 4:**
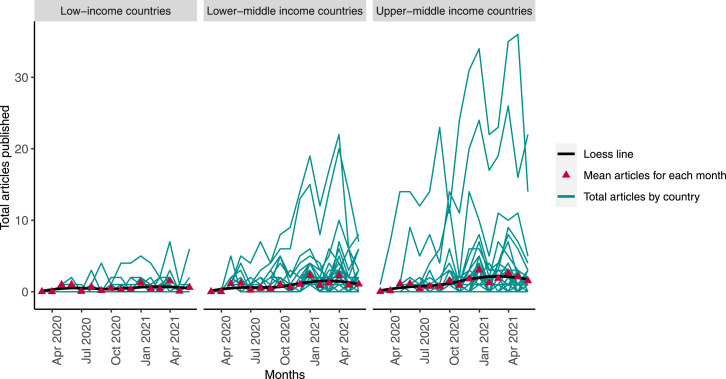
Total publications related to COVID-19 and women's well-being including focus on a given LMIC, every month between Feb 2020 and May 2021, by income type: low- income country, lower-middle income country, and upper- middle income country Note: *Each line indicates temporal trends in publications for each country included in the study sample*.

The number of women's well-being related COVID-19 articles published by May 2021 including focus on LMICs generally increased over time (Figure 3). On average over this time period, there were 67 women's well-being related publications per month (media *n=*79), though these trends also varied by country income group, with greater representation of upper-middle income countries than lower-income countries (Figure 4). China had more publications related to COVID-19 and women's well-being than any other country in this review, with 315 articles indexed between February 2020 and May 2021.

In multivariable linear mixed effect modelling, the number of COVID-19 cases per 100 population in a country was significantly and positively associated with the number of women's well-being related COVID-19 academic publications during our study period. For every one unit increase in the natural log number of COVID-19 cases per 100 people in a country in a given month, there was a 0·2 unit increase in the natural log of women's well-being related COVID-19 publications in that country six months later (*p*<0·001) (Table 3). COVID-19 deaths per 100 population were only marginally significantly related to the natural log number of publications related to COVID-19 and women's well-being in a country. However, for every one unit increase in the natural log of the COVID-19 case fatality ratio, there was a 0·05 increase in the natural log of publications related to COVID-19 and women's well-being six months later (*p*<0·001).

**Table 3:** Linear mixed effect models examining the relationship between COVID-19 burden in a country and the natural log of the number of publications related to COVID-19 and women's well-being including focus on that country six months later.

	Estimate	SE	*p*-value	Estimate	SE	*p*-value	Estimate	SE	*p*-value
*Within effects*									
ln COVID-19 cases per 100 population	0·17	0·05	<0·001						
ln COVID-19 deaths per 100 population				2·89	1·49	0·05			
ln COVID-19 case fatality ratio							0·05	0·01	<0·001
Author affiliations with focal country									
Neither lead nor senior	0·26	0·01	<0·001	0·26	0·01	<0·001	0·25	0·01	<0·001
Only lead	0·16	0·04	<0·001	0·17	0·04	<0·001	0·16	0·04	<0·001
Only senior	0·32	0·05	<0·001	0·32	0·05	<0·001	0·32	0·05	<0·001
Both lead and senior/Lead author in single-author paper	0·08	0·01	<0·001	0·09	0·01	<0·001	0·09	0·01	<0·001
*Between effects*									
ln COVID-19 cases per 100 population (mean)	0·23	0·12	0·06						
ln COVID-19 deaths per 100 population (mean)				0·90	2·94	0·76			
ln COVID-19 case fatality ratio (mean)							0·02	0·02	0·47
Author affiliations with focal country									
Neither lead nor senior (mean)	0·51	0·04	<0·001	0·52	0·04	<0·001	0·53	0·04	<0·001
Only lead (mean)	0·73	0·16	<0·001	0·72	0·17	<0·001	0·73	0·18	<0·001
Only senior (mean)	0·08	0·29	0·77	0·09	0·29	0·77	0·05	0·32	0·87
Both lead and senior/Lead author in single-author paper (mean)	0·00	0·01	0·83	0·00	0·02	1·00	-0·00	0·02	0·83
Income group									
Low	REF			REF			REF		
Lower-middle	0·01	0·03	0·87	0·01	0·03	0·84	0·01	0·04	0·78
Upper-middle	-0·02	0·04	0·69	0·01	0·04	0·87	0·01	0·04	0·86
Gender Inequality Index	0·12	0·10	0·22	0·07	0·10	0·51	0·05	0·11	0·66
ln development assistance (USD, millions)	0·02	0·02	0·32	0·01	0·02	0·43	0·01	0·02	0·58
*Random effects*									
Country		0·08			0·08			0·09	
Residual		0·30			0·30			0·30	
*Model fit*									
AIC	821·84			819·56			809·52		

Note: Outcome is natural log of the number of publications.

All assessed author affiliation categories were significantly and positively associated with the number of publications related to COVID-19 and women's well-being in models adjusting for COVID-19 cases, deaths and case-fatality ratio (Table 3). In all models, however, effect sizes were largest for senior author in-country affiliations, followed by neither lead nor senior author in-country affiliations, then lead author only affiliations, and finally lead and senior author in-country affiliations.

Income-group stratified analyses indicate that the positive association between COVID-19 burden and women's well-being related publications six months later was significant only in upper-middle income countries (cases coefficient=0·3, *p*<0·001 [Appendix Table 3], deaths coefficient=5·0, *p*<0·001 [Appendix Table 4], case fatality ratio coefficient=0·1, *p*<0·001 [Appendix Table 5]). Authorship remained significantly and positively associated with publications in all income groups barring in-country affiliated lead authorship in upper-middle income countries (Appendix Table 3, Table 4, Table 5).

**Table 4:** Linear mixed effect models examining the relationship between COVID-19 cases in a country and the number of publications related to COVID-19 and women's well-being focused women and girls’ health, gendered social outcomes, and gendered economic impacts including focus on that country six months later.

	Women and girls’ health			Gendered social outcomes			Gendered economic impacts		
	Estimate	SE	*p*-value	Estimate	SE	p-value	Estimate	SE	*p*-value
*Within effects*									
ln COVID-19 cases per 100 population	-0·17	0·07	0·02	0·13	0·07	0·08	-0·01	0·05	0·85
Author affiliations with focal country									
Neither lead nor senior	0·83	0·01	<0·001	0·12	0·01	<0·001	0·09	0·01	<0·001
Only lead	1·04	0·05	<0·001	0·14	0·05	0·01	-0·06	0·04	0·08
Only senior	0·79	0·06	<0·001	0·23	0·07	<0·001	-0·02	0·05	0·60
Both lead and senior/Lead author in single-author paper	0·94	0·01	<0·001	0·12	0·01	<0·001	0·01	0·01	0·20
*Between effects*									
ln COVID-19 cases per 100 population (mean)	-0·08	0·12	0·67	0·12	0·13	0·06	-0·03	0·08	0·76
Author affiliations with focal country									
Neither lead nor senior (mean)	0·68	0·04	<0·001	0·32	0·04	<0·001	0·19	0·03	<0·001
Only lead (mean)	1·08	0·16	<0·001	-0·09	0·18	0·61	0·11	0·11	0·34
Only senior (mean)	0·68	0·28	0·01	0·20	0·31	0·51	0·15	0·20	0·43
Both lead and senior/Lead author in single-author paper (mean)	1·06	0·01	<0·001	-0·03	0·02	0·03	-0·06	0·01	<0·001
Income group									
Low	REF			REF			REF		
Lower-middle	0·03	0·03	0·30	-0·00	0·03	0·93	0·02	0·02	0·25
Upper-middle	0·08	0·04	0·06	-0·01	0·05	0·79	0·01	0·03	0·75
Gender Inequality Index	0·01	0·02	0·95	0·11	0·11	0·31	0·06	0·07	0·41
ln development assistance (USD, millions)	-0·01	0·02	0·51	0·02	0·02	0·19	0·01	0·01	0·28
*Random effects*									
Country		0			0·04			0·00	
Residual		0·41			0·42			0·29	
*Model fit*									
AIC	1763·4			1873·5			640·5

**Table 5:** Linear mixed effect models examining the relationship between COVID-19 deaths in a country and the number of publications related to COVID-19 and women's well-being focused women and girls’ health, gendered social outcomes, and gendered economic impacts including focus on that country six months later.

	Women and girls’ health			Gendered social outcomes			Gendered economic impacts		
	Estimate	SE	*p*-value	Estimate	SE	p-value	Estimate	SE	*p*-value
*Within effects*									
ln COVID-19 deaths per 100 population	-3·76	2·04	0·07	7·69	2·10	<0·001	1·15	1·44	0·42
Author affiliations with focal country									
Neither lead nor senior	0·83	0·01	<0·001	0·12	0·01	<0·001	0·09	0·01	<0·001
Only lead	1·04	0·05	<0·001	0·14	0·05	0·01	-0·07	0·04	0·07
Only senior	0·79	0·06	<0·001	0·23	0·07	<0·001	-0·02	0·05	0·59
Both lead and senior/Lead author in single-author paper	0·94	0·01	<0·001	0·12	0·01	<0·001	0·01	0·01	0·21
*Between effects*									
ln COVID-19 deaths per 100 population (mean)	2·19	2·77	0·43	-1·95	3·09	0·53	-0·62	1·95	0·75
Author affiliations with focal country									
Neither lead nor senior (mean)	0·66	0·04	<0·001	0·34	0·04	<0·001	0·19	0·03	<0·001
Only lead (mean)	1·08	0·16	<0·001	-0·09	0·18	0·61	0·11	0·11	0·33
Only senior (mean)	0·66	0·28	0·02	0·22	0·31	0·47	0·16	0·20	0·42
Both lead and senior/Lead author in single-author paper (mean)	1·07	0·02	<0·001	-0·04	0·02	0·02	-0·06	0·01	<0·001
Income group									
Low	REF			REF			REF		
Lower-middle	0·03	0·03	0·29	-0·00	0·03	0·92	0·02	0·02	0·26
Upper-middle	0·06	0·04	0·11	0·01	0·04	0·90	0·01	0·03	0·78
Gender Inequality Index	0·05	0·09	0·61	0·06	0·11	0·59	0·06	0·07	0·39
ln development assistance (USD, millions)	-0·01	0·02	0·69	0·02	0·02	0·29	0·01	0·01	0·30
*Random effects*									
Country		0			0·05			0	
Residual		0·41			0·42			0·29	
*Model fit*									
AIC	1752·3			1850·6			626·8		

Analyses exploring the relationship between our independent variables and specific topics of focus within publications related to COVID-19 and women's well-being reveal more variable associations. The number of COVID-19 cases per 100 population in a country was significantly and negatively associated with the number of women's well-being related COVID-19 academic publications focused on women and girls’ health during our study period (coefficient=-0·17, *p*=0·02)(Table 4). In contrast, the number of COVID-19 deaths per 100 population was significantly and positively associated with academic publications on gendered social outcomes (coefficient=7·7, *p*<0·001) (Table 5). There was no statistical association between COVID-19 case fatality ratio and publications focused on women and girls’ health, gendered social outcomes, or gendered economic impacts (Table 6). Having in-country affiliated lead and/or senior authors was associated with publications in women and girls’ health and gendered social outcomes, but not gendered economic impacts (Table 4, Table 5, Table 6).

**Table 6:** Linear mixed effect models examining the relationship between COVID-19 case fatality ratio in a country and the number of publications related to COVID-19 and women's well-being focused women and girls’ health, gendered social outcomes, and gendered economic impacts including focus on that country six months later.

	Women and girls’ health			Gendered social outcomes			Gendered economic impacts		
	Estimate	SE	*p*-value	Estimate	SE	*p*-value	Estimate	SE	*p*-value
*Within effects*									
ln COVID-19 case fatality ratio	0·02	0·02	0·29	0·02	0·02	0·37	0·01	0·01	0·41
Author affiliations with focal country									
Neither lead nor senior	0·83	0·01	<0·001	0·12	0·01	<0·001	0·09	0·01	<0·001
Only lead	1·04	0·05	<0·001	0·14	0·06	0·01	-0·07	0·04	0·08
Only senior	0·80	0·07	<0·001	0·23	0·07	<0·001	-0·03	0·05	0·59
Both lead and senior/Lead author in single-author paper	0·93	0·01	<0·001	0·12	0·01	<0·001	0·01	0·01	0·13
*Between effects*									
ln COVID-19 case fatality ratio (mean)	0·05	0·02	0·03	-0·06	0·03	0·03	-0·02	0·02	0·21
Author affiliations with focal country									
Neither lead nor senior (mean)	0·66	0·04	<0·001	0·36	0·04	<0·001	0·20	0·03	<0·001
Only lead (mean)	1·06	0·17	<0·001	-0·06	0·18	0·72	0·13	0·12	0·27
Only senior (mean)	0·71	0·29	0·02	0·16	0·32	0·62	0·13	0·21	0·53
Both lead and senior/Lead author in single-author paper (mean)	1·07	0·01	<0·001	-0·05	0·02	<0·001	-0·07	0·01	<0·001
Income group									
Low	REF			REF			REF		
Lower-middle	0·05	0·03	0·17	-0·01	0·04	0·69	0·02	0·02	0·32
Upper-middle	0·08	0·04	0·06	-0·00	0·05	0·93	0·01	0·03	0·85
Gender Inequality Index	0·01	0·10	0·92	0·11	0·11	0·33	0·08	0·07	0·25
ln development assistance (USD, millions)	-0·01	0·02	0·49	0·03	0·02	0·16	0·01	0·01	0·22
*Random effects*									
Country		0·00			0·03			0·00	
Residual		0·42			0·44			0·30	
*Model fit*									
AIC	1738·3			1846·6			721·41		

## Discussion

This bibliometric analysis of publications related to COVID-19 and women's well-being identified more than 1,000 peer-reviewed articles published between February 2020 and May 2021, exploring ways that gender and COVID-19 have intersected in LMICs. This work documents gendered impacts of the pandemic with regard to health as well as social and economic concerns in LMIC contexts, though the social and economic impacts have received less attention. Importantly, our analysis highlights that publication numbers on this topic were generally reflective of COVID-19 burden, but this was limited to upper-middle income nations, suggesting that lower-middle and low-income countries may be under-represented in our developing understanding of gender and the pandemic. Our findings correspond with data showing that pandemic-related publications were generally produced by authors in the most affected countries, though this early work was largely concentrated in upper-middle and high-income nations.^47^

There were important distinctions in the topical foci of publications related to COVID-19 and women's well-being based on the nature of COVID-19 case burdens. Increases in COVID-19 cases in LMICs were associated with decreases in publications focused on women and girls’ health, while increases in COVID-19 deaths were associated with increases in publications on gendered social outcomes, including social norms. Nonetheless, we found no association between case fatality ratios and publications. Case fatality ratios represent a broader range of structural inequities and vulnerabilities, including health system access and infrastructure, and are thus a meaningful lens through which to examine pandemic response.^48^, ^49^, ^50^, ^51^, ^52^ Lack of effects may again point to infection burden indicators not being sufficient to build research response in low and strained resource contexts. Hence, we may least understand the gendered impacts of the pandemic in the most socially and economically vulnerable nations.

Upper-middle income countries are most represented in this body of literature, influenced in particular by China, which was a focal country in more than one in three articles published during this period. As the country where the first COVID-19 outbreak was identified,^53^ this volume of research represents a rapid production of research aimed at understanding the characteristics and manifestations of this virus, despite a low level of population-adjusted COVID-19 cases and deaths, relative to many other countries. However, the association between COVID cases and women's well-being related COVID publications in upper-middle income countries was present even after excluding China (results not shown), indicating that this relationship is reflective of a broader spectrum of characteristics of upper-middle income countries. Upper-middle income countries tend to have stronger existing data infrastructures than lower-income counterparts, and thus an ability to more rapidly pivot epidemiologic data systems, including well-functioning Civil Registry and Vital Statistics systems, to capture information on COVID-19. Indeed, the World Health Organization found that 72% of upper-middle income countries had well-developed or sustainable capacities to survey public health threats, in comparison to only 41% of low-income countries.^54^ Countries with less robust existing infrastructures had less capacity to track, identify and report COVID-19 cases and deaths through national statistical offices, particularly early on in the pandemic, and there were pronounced differences across income strata.^55^ Responsive gender data systems able to pivot across data collection modalities and track key data are lacking in many countries, and a key area for augmentation moving forward.^6^

Research leadership from a more diverse array of scientists, particularly those from LMICs, is widely recognized as an area in need of expansion.^18^^,^^31^^,^^56^^,^^57^ While collaboration is an important factor in global health research, including COVID-19 research, leadership by authors in LMICs is needed to appropriately reflect local context in the interpretation of research results, as well as to address pervasive power imbalances.^31^^,^^57^, ^58^, ^59^, ^60^ To that end, this study offers important support. We find that the majority of both lead and senior authors had their primary institutional affiliation within a focal country of their research article. Further, such authorship positioning for in-country authors was associated with a higher number of women's well-being and COVID-19 publications. However, one in eight papers still had neither lead nor senior author affiliated with an in-country institution, and this affects the focus of the women's well-being and COVID-19 papers produced. We found that papers with neither lead nor senior authors from within the LMIC context of the research produced half or more of articles focused on gendered economic impacts and women's leadership. Our linear mixed effect models indicate that having neither lead nor senior authors affiliated with in-country institutions had an approximately 2·5 times large coefficient size for the number of publications related to COVID-19 and women's well-being than having both lead and senior authors affiliated with in-country institutions.

Having a body of researchers in-country able to produce published research on topics such as the gendered effects of COVID-19 is critical to ensure that governments, aid organizations, multilateral and donor organizations and scientists can learn more about the effects of this pandemic and design appropriately targeted and responsive policies, programs and future research endeavours. These levels of affiliation with in-country institutions are similar to those seen in prior research,^18^ but the sharp drops in in-country affiliated authors in leadership positions for scientific research on gendered social outcomes, and gendered economic impacts highlight ongoing gaps in representation. Prior research shows that COVID-19 publications are more than thrice as likely to focus on health rather than social issues,^27^ an understandable prioritization given that this is an infectious disease. It is, however, worrisome that LMIC-affiliated leadership authors are under-represented in research production on gendered social impacts, given their likely greater understanding of the social context of findings than researchers from outside of that nation. LMIC-affiliated authors may also face more barriers in the production of peer-reviewed publications due to the potentially prohibitively high publication costs in peer-reviewed biomedical and public health journals.^61^^,^^62^ Further research is needed to determine what level of these gaps in in-country author leadership for social and economic gender research production are caused by inadequate numbers of scholars interested in this field, a lack of capacity, gaps in in-country tertiary education, limited funding for research or publication costs, or other factors.

Development assistance was not associated with publications related to COVID-19 and women's well-being in any income group, or in any focal topic. This may in part be due to the fact that these figures represent total development assistance; only 4% of these monies are dedicated primarily to gender programming, and a smaller portion still is dedicated to gendered health responses.^63^ Low-income countries received less absolute development assistance funding than lower-middle or upper-middle countries in this sample (an average of USD$ 1·97 million per low-income country in this sample in 2019 vs. an average of USD$2·63 million per lower-middle income country and USD$2·57 million per upper-middle income country). These lower levels of development assistance, paired with substantially higher average levels of gender inequalities in lower-income countries, echo the well-recognized need for increased investment in gender programming, research, and resources in lower-income countries.^64^ The research to publication pipeline is intensive in terms of intellectual, human and financial resources, as well as specialized expertise for gender-focused research;^65^^,^^66^ these disparities may be jointly hindering the production of women's well-being related COVID-19 research in low-income countries.

Study findings must be interpreted in light of known limitations. This is an ecological, bibliometric analysis of English-language publications, and is neither able to make causal assumptions nor able to draw conclusions about individual countries or health systems. It is possible that some literature related to women's well-being and COVID-19 was not identified using the study search terms, though we attempted to be as comprehensive as possible. Further, consideration of the impact of non-empirical pieces was outside of the scope of the analysis and may merit further review elsewhere. Our data on COVID-19 cases and deaths relies on numbers reported to the World Health Organization, and thus represents primarily lab-confirmed cases and deaths, not total cases and deaths.^1^ COVID-19 excess mortality is now being estimated using statistical modelling techniques, efforts to date primarily target deaths, rather than cases, and thus do not allow for estimation of all outcomes used in this analysis.^67^ In addition, data on COVID-19 cases and deaths was not available disaggregated by sex, a known data gap.^68^ Importantly, studies needed to include gendered findings to be eligible for inclusion in this analysis, but gender did not need to be the primary analytic aim, thus these papers do not represent comprehensive gender analyses. While women's well-being related publications should include perspectives of gender beyond the binary man/woman, this review identified only two studies that focused on gendered social outcomes for transgender persons; this limited sample size precluded detailed analysis of this population. Finally, our measure of lead and senior author affiliations is limited to their institutional affiliations at the time of article publication. This does not necessarily correspond with their nationality, or even long-term residence, and should not be interpreted as such.

The COVID-19 pandemic has adversely impacted the lives of millions of people around the world, and research has been foundational in understanding the health, social and economic consequences of crisis. Many of these consequences manifest in gendered ways, and understanding these differential manifestations is critical to informing responsive recovery and support policies and programs. Our findings generally found higher levels of publications related to COVID-19 and women's well-being in countries with higher levels of population-adjusted cases and deaths, an encouraging finding that many scientists, funders and governments are prioritizing understanding the gender-related lessons of this pandemic. However, these relationships were significant only among upper-middle income countries, and varied by specific gender focal topic. We also found that in-country affiliated authorship is associated with more women's well-being and COVID-19 publications, but disproportionately limited to health. In fact, lower levels of representation of in-country affiliated authorship in leadership positions was associated with generation of papers focusing on social, economic, and political impacts of the pandemic. Gender-intentional lenses are an essential aspect of COVID-19 response plans in all countries, and should be prioritized by governments, funders, and researchers alike. Strong leadership by in-country scientists inclusive of social and behavioural scientists within health research, as well as flexible, responsive gender data systems are foundational. Application of these lenses and research led by local scholars should be bolstered in low resource settings, or we will continue to have inadequate understanding of gendered health impacts in these contexts.

## Contributors

LM, ND and AR contributed to study design and conceptualization. LM, ND, WWC and AD contributed to data collection. LM and ND contributed to data analysis and figure and table creation. LM wrote the original manuscript, all authors contributed to manuscript review and editing.

## Data sharing statement

No individually identifiable information is included in the studies included in this review. All extracted data have been made publicly available at Mendeley Data (doi: 10.17632/5hfcxsbstk.1).

## Declaration of interests

All authors report funding support in the form of a grant from the Bill and Melinda Gates Foundation to their institution to conduct this research (PI: AR). All authors have nothing else to disclose.

## Appendix Supplementary materials

Image, application 1
