# Supplementary material for: COVID-19 burden, author affiliation and women's well-being: A bibliometric analysis of COVID-19 related publications including focus on low- and middle-income countries
Source: eClinicalMedicine. 2022 Aug 3;52:101606. doi: 10.1016/j.eclinm.2022.101606 (PMC9347236; doi:10.1016/j.eclinm.2022.101606)
Supplement: Supplementary file 1 [file mmc1.pdf]

COVID-19 burden, author affiliation and women's well-being: A bibliometric analysis of COVID-19 related publications including focus on low- and middle-income countries

Lotus McDougal, Nabamallika Dehingia, Wendy Wei Cheung, Anvita Dixit, Anita Raj

Supplementary material contents

Appendix Table 1. Search criteria for identifying articles on COVID-19 and women's well-being. 2

Appendix Table 2. Country-level data used in the study 3

Appendix Figure 1. Country income group-stratified scatterplots of the natural log of cumulative of publications related to COVID-19 and women's well-being with focus on low and middle countries and the natural log of cumulative COVID-19 cases per 100 individuals (A), deaths per 100 population (B), and case fatality ratios (C) over the study period. 8

Appendix Table 3. Country income group-stratified linear mixed effect models examining the relationship between COVID-19 cases per 100 population in a country and the number of publications related to COVID-19 and women's well-being including focus on that country six months later. 9

Appendix Table 4. Country income group-stratified linear mixed effect models examining the relationship between COVID-19 deaths per 100 population in a country and the number of publications related to COVID-19 and women's well-being including focus on that country six months later. 10

Appendix Table 5. Country income group-stratified linear mixed effect models examining the relationship between COVID-19 case fatality ratio in a country and the number of publications related to COVID-19 and women's well-being including focus on that country six months later 11

Appendix Table 1. Search criteria for identifying articles on COVID-19 and women's well-being.

Thematic area	Search terms
Women and girls' health	(covid OR coronavirus OR SARS-CoV-2) AND<br>(gender OR women OR woman) AND<br>(maternal OR pregnant OR birth OR antenatal OR reproductive OR sexual OR "family planning" OR psychological OR mental OR anxiety OR stress OR menstrual OR "health worker" Or nurse OR midwife OR knowledge OR information)
Gender norms and gendered social outcomes	(covid OR coronavirus OR SARS-CoV-2) AND<br>(gender OR women OR woman) AND<br>(freedom OR coercion OR agency OR empower OR marriage OR violence OR access OR media OR unpaid OR domestic OR household OR trafficking OR exploitation OR "digital inclusion" OR "gender norms" OR "gender roles" OR "child care")
Gendered economic impacts	(covid OR coronavirus OR SARS-CoV-2) AND<br>(gender OR women OR woman) AND<br>(collective OR economy OR "financial inclusion" OR money OR "food insecurity" OR loan OR borrow OR asset OR bank OR saving OR poverty OR market OR "government scheme" OR "financial autonomy" OR enterprise OR business OR "informal work")
Women's leadership	(covid OR coronavirus OR SARS-CoV-2) AND<br>(gender OR women OR woman) AND<br>(leader OR manager OR supervisor OR elected)
Women's collectives	(covid OR coronavirus OR SARS-CoV-2) AND<br>(gender OR women OR woman) AND<br>(collective OR "women's group" OR "women's collective" OR "participatory group")

Appendix Table 2. Country-level data used in the study

Country	Total publications	Income group[1]	Cumulative COVID-19 cases[2]	Cumulative COVID-19 deaths[2]	Population (2019)	Total development assistance received (2019)	Gender Inequality Index (2019)
Afghanistan	9	LI	71838	2944	38041754	$4,156,356,806	0·655
Albania	13	UMI	132309	2450	2854191	$327,388,228	0·181
Algeria	7	LMI	128725	3465	43053054	-$21,094,394	0·429
Angola	6	LMI	34366	764	31825295	$83,142,034	0·536
Argentina	25	UMI	3732263	77108	44938712	$3,480,839,024	0·328
Armenia	4	UMI	222670	4438	2957731	$750,023,495	0·245
Azerbaijan	4	UMI	333864	4907	10023318	$944,801,299	0·323
Bangladesh	42	LMI	800540	12619	163046161	$5,921,985,910	0·537
Belarus	6	UMI	393807	2841	9466856	$519,285,443	0·118
Belize	4	LMI	12791	324	390353	$ 61,454,050	0·415
Benin	5	LMI	8058	101	11801151	$350,478,749	0·612
Bhutan	3	LMI	1620	1	763092	$185,066,161	0·421
Bolivia	11	LMI	366714	14435	11513100	$1,108,606,950	0·417
Bosnia and Herzegovina	7	UMI	203978	9215	3301000	$701,785,880	0·149
Botswana	6	UMI	56313	831	2303697	-$74,843,213	0·465
Brazil	93	UMI	16471600	461057	211049527	$ 10,183,529,490	0·408
Bulgaria	6	UMI	418274	17662	6975761	#N/A	0·206
Burkina Faso	8	LI	13430	166	20321378	$1,177,858,655	0·594
Burundi	5	LI	4790	6	11530580	$667,006,126	0·504
Cabo Verde	4	LMI	30359	264	549935	$183,093,936	0·397
Cambodia	5	LMI	30093	214	16486542	$1,696,849,469	0·474
Cameroon	9	LMI	77982	1270	25876380	$1,575,758,869	0·56
Central African Republic	4	LI	7085	98	4745185	$607,000,251	0·68
Chad	7	LI	4929	173	15946876	$655,534,937	0·71
China	315	UMI	101426	4742	1397715000	$ 42,378,942,490	0·168
Colombia	27	UMI	3363061	87747	50339443	$3,606,024,088	0·428

Comoros	4	LMI	3951	146	850886	$ 73,479,623	-
Congo	4	LMI	11658	153	5380508	-$442,655,003	0·57
Costa Rica	9	UMI	316440	3993	5047561	$673,043,111	0·288
Cote d'Ivoire	6	LMI	47195	301	25716544	$1,759,844,157	0·638
Cuba	10	UMI	141166	950	11333483	$494,667,009	0·304
Democratic People's Republic of Korea	0	LI	0	0	25778815	-	-
Democratic Republic of the Congo	12	LI	31651	782	86790567	$3,256,366,708	0·617
Djibouti	5	LMI	11528	154	973560	$303,502,979	-
Dominica	2	UMI	188	0	71808	$ 42,990,049	-
Dominican Republic	11	UMI	291910	3628	10738958	$1,770,268,071	0·455
Ecuador	18	UMI	425841	20545	17373662	$2,823,652,478	0·384
Egypt	26	LMI	261666	15047	100388073	$5,318,953,623	0·449
El Salvador	6	LMI	73246	2245	6453553	$1,180,647,612	0·383
Equatorial Guinea	4	UMI	8529	118	1355986	$ 55,239,987	0
Eritrea	4	LI	4061	14	3213972	$264,699,473	-
Eswatini	4	LMI	18591	673	1148130	$104,203,277	0·567
Ethiopia	42	LI	271345	4155	112078730	$4,793,374,195	0·517
Fiji	2	UMI	401	4	889953	$148,859,771	0·37
Gabon	5	UMI	24365	150	2172579	$313,310,233	0·525
Gambia	5	LI	5993	179	2347706	$279,911,199	0·612
Georgia	6	UMI	343963	4773	3720382	$1,127,878,433	0·331
Ghana	17	LMI	93898	785	30417856	$1,127,631,618	0·538
Grenada	3	UMI	161	1	112003	$ 17,586,699	-
Guatemala	8	UMI	254225	8137	16604026	$418,872,916	0·479
Guinea	5	LI	23172	161	12771246	$881,755,694	-
Guinea-Bissau	4	LI	3766	68	1967998	#N/A	-
Guyana	4	UMI	16822	382	782766	$1,476,804,982	0·462
Haiti	6	LMI	14711	319	11263077	$730,037,931	0·636
Honduras	16	LMI	236952	6296	9746117	$1,003,298,738	0·423
India	136	LMI	28047529	329100	1366417754	$ 24,001,930,050	0·488

Indonesia	25	LMI	1821703	50578	270625568	$ 14,966,289,450	0·48
Iran	116	LMI	2902094	79939	82913906	-$227,024,837	0·459
Iraq	17	UMI	1197082	16351	39309783	$920,123,200	0·577
Jamaica	5	UMI	48467	945	2948279	$215,920,935	0·396
Jordan	33	UMI	735811	9452	10101694	$3,834,293,443	0·45
Kazakhstan	6	UMI	442935	7321	18513930	-$1,023,305,581	0·19
Kenya	19	LMI	170647	3157	52573973	$3,169,645,388	0·518
Kiribati	3	LMI	0	0	119446	-	-
Kosovo	4	UMI	107362	2233	1794248	$482,897,775	-
Kyrgyzstan	4	LMI	104729	1808	6456900	$474,449,090	0·369
Lao	4	LMI	1851	3	7169455	$739,409,656	0·459
Lebanon	16	UMI	540277	7723	6855713	$1,212,487,863	0·411
Lesotho	5	LMI	10831	326	2125268	$122,526,985	0·553
Liberia	5	LI	2179	86	4937374	$802,972,714	0·65
Libya	8	UMI	185181	3125	6777452	$778,699,910	0·252
Madagascar	6	LI	41266	835	26969307	$1,181,310,692	-
Malawi	10	LI	34335	1155	18628747	$1,156,106,777	0·565
Malaysia	21	UMI	565525	2729	31949777	$ 11,070,760,360	0·253
Maldives	6	UMI	64396	161	530953	$212,051,195	0·369
Mali	6	LI	14265	517	19658031	$1,847,690,933	0·671
Marshall Islands	1	UMI	4	0	58791	$240,419,265	-
Mauritania	4	LMI	19494	463	4525696	-$342,533,694	0·634
Mauritius	2	UMI	1393	17	1265740	#N/A	0·347
Mexico	53	UMI	2411503	223455	127575529	$ 13,313,689,500	0·322
Micronesia	2	LMI	0	0	113815	-$1,122,200,322	-
Mongolia	5	LMI	57512	272	3225167	$1,321,478,609	0·322
Montenegro	4	UMI	99606	1583	622137	$216,452,577	0·109
Morocco	8	LMI	519108	9143	36471769	$2,868,946,967	0·454
Mozambique	8	LI	70780	836	30366036	$2,157,860,013	0·523
Myanmar	8	LMI	143629	3217	54045420	$2,730,845,900	0·478

Namibia	4	UMI	55141	824	2494530	$150,571,029	0·44
Nepal	29	LMI	561297	7386	28608710	$1,413,546,737	0·452
Nicaragua	4	LMI	5833	186	6545502	$415,999,232	0·428
Niger	5	LI	5410	192	23310715	$1,446,630,393	0·642
Nigeria	42	LMI	166315	2071	200963599	$6,383,572,786	-
North Macedonia	10	UMI	155269	5408	2083459	$473,441,648	0·143
Pakistan	54	LMI	918936	20736	216565318	$2,863,222,979	0·538
Panama	5	UMI	377428	6369	4314768	-	0·407
Papua New Guinea	4	LMI	15910	162	8776109	$528,357,907	0·725
Paraguay	9	UMI	350613	8987	7044636	$214,672,273	0·446
Peru	44	UMI	1951651	183470	32510453	$609,693,027	0·395
Philippines	16	LMI	1223616	20860	1·08E+08	$ 32,887,351,520	0·43
Republic of Moldova	3	UMI	255145	6104	2657637	$461,538,589	0·204
Romania	15	UMI	1077584	33863	19286123	-	0·276
Russian Federation	18	UMI	5071915	121501	144373535	-	0·225
Rwanda	10	LI	26918	352	12626950	$1,309,834,949	0·402
Saint Lucia	4	UMI	5053	77	182790	$ 52,227,310	0·401
Saint Vincent and The Grenadines	3	UMI	2035	12	110589	$ 82,080,277	-
Samoa	6	UMI	1	0	197097	$121,279,192	0·36
Sao Tome and Principe	3	LMI	2345	37	215056	$ 49,959,548	0·537
Senegal	8	LMI	41387	1138	16296364	$1,999,151,489	0·533
Serbia	11	UMI	712224	6854	6944975	$1,983,381,570	0·132
Sierra Leone	8	LI	4140	79	7813215	$730,499,977	0·644
Solomon Islands	3	LMI	20	0	669823	$299,763,085	-
Somalia	6	LI	14660	769	15442905	$1,718,680,608	-
South Africa	31	UMI	1662825	56439	58558270	$5,239,323,542	0·406
South Sudan	5	LI	10688	115	11062113	$1,684,866,763	-
Sri Lanka	13	LMI	186359	1484	21803000	$581,031,778	0·401
Sudan	7	LI	35495	2630	42813238	$1,505,292,218	0·545
Suriname	6	UMI	14515	288	581363	$251,565,852	0·436

Syrian Arab Republic	9	LI	24467	1766	17070135	$ 10,110,941,450	0·482
Tajikistan	3	LMI	13714	91	9321018	$417,924,624	0·314
Tanzania	8	LMI	509	21	58005463	$1,944,754,565	0·556
Thailand	16	UMI	159769	1031	69625582	$7,843,365,222	0·359
Timor-Leste	3	LMI	6994	16	1293119	$273,269,754	-
Togo	7	LI	13457	125	8082366	$333,427,832	0·573
Tonga	3	UMI	0	0	105697	-	0·354
Tunisia	11	LMI	344688	12623	11694719	$1,635,134,089	0·296
Turkey	196	UMI	5242911	47405	83429615	$ 11,557,631	0·306
Turkmenistan	3	UMI	0	0	6031187	-	-
Tuvalu	1	UMI	0	0	11792	-	-
Uganda	17	LI	47147	362	44269594	$2,374,579,381	0·535
Ukraine	7	LMI	2202494	50536	44385155	$3,329,710,562	0·234
Uzbekistan	4	LMI	100335	690	33580650	$3,578,635,380	0·288
Vanuatu	4	LMI	3	0	299882	$142,991,153	-
Venezuela	10	UMI	462886	5230	28515829	-$290,902,718	0·479
Vietnam	23	LMI	7166	47	96462106	$8,056,017,118	0·296
West Bank and Gaza	14	LMI	336889	3763	4685306	$2,297,447,351	-
Yemen	4	LI	6737	1320	29161922	$4,043,176,541	0·795
Zambia	8	LMI	95050	1278	17861030	$958,723,637	0·539
Zimbabwe	13	LMI	38944	1594	14645468	$781,765,184	0·527

[1] source: World Bank 2021-2022 classification. LI=Low-income, LMI=Lower-middle income, UMI=Upper-middle income

[2] source: WHO Coronavirus Dashboard, through May 31st 2021

A.

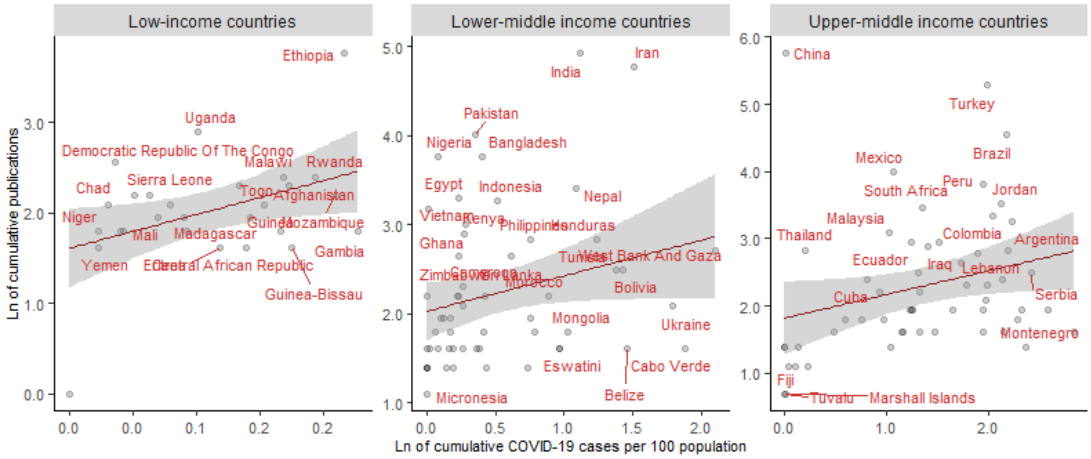

B.

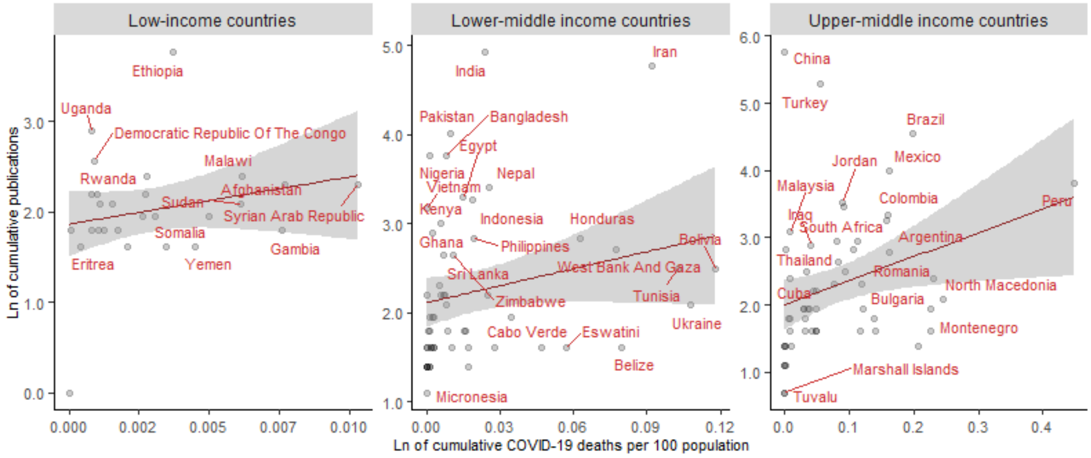

C.

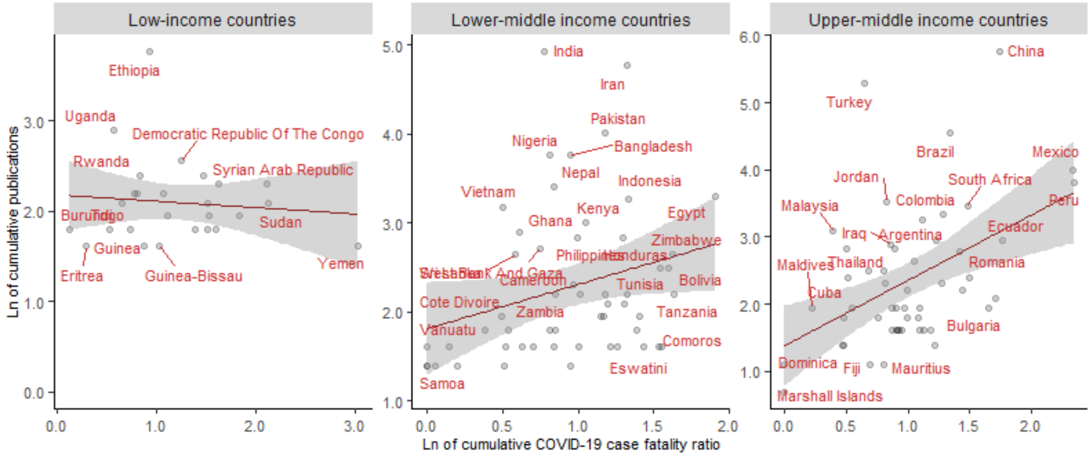

Appendix Figure 1. Country income group-stratified scatterplots of the natural log of cumulative of publications related to COVID-19 and women's well-being with focus on low and middle countries and the natural log of cumulative COVID-19 cases per 100 individuals (A), deaths per 100 population (B), and case fatality ratios (C) over the study period.

Appendix Table 3. Country income group-stratified linear mixed effect models examining the relationship between COVID-19 cases per 100 population in a country and the number of publications related to COVID-19 and women's well-being including focus on that country six months later.

	Low-income			Lower-middle income			Upper-middle income		
	Estimate	SE	p-value	Estimate	SE	p-value	Estimate	SE	p-value
Within effects									
ln COVID-19 cases per 100 population	-0·37	0·64	0·56	-0·10	0·13	0·41	0·27	0·06	<0·001
Author affiliations with focal country									
Neither lead nor senior	0·55	0·01	<0·001	0·29	0·01	<0·001	0·21	0·01	<0·001
Only lead	0·41	0·05	<0·001	0·34	0·05	<0·001	-0·02	0·06	0·82
Only senior	0·44	0·05	<0·001	0·42	0·07	<0·001	0·31	0·07	<0·001
Both lead and senior/Lead author in single-author paper	0·32	0·02	<0·001	0·13	0·01	<0·001	0·07	0·01	<0·001
Between effects									
ln COVID-19 cases per 100 population (mean)	1·04	0·14	0·36	0·07	0·27	0·81	0·21	0·13	0·21
Author affiliations with focal country									
Neither lead nor senior (mean)	0·48	0·07	<0·001	0·33	0·06	<0·001	0·60	0·06	<0·001
Only lead (mean)	1·24	0·49	0·01	0·86	0·23	<0·001	-0·39	0·32	0·23
Only senior (mean)	0·02	0·25	0·94	0·54	0·70	0·45	0·18	0·36	0·63
Both lead and senior/Lead author in single-author paper (mean)	0·13	0·09	0·15	0·11	0·04	0·02	0·00	0·02	0·81
Gender Inequality Index	-0·24	0·11	0·03	0·11	0·16	0·50	0·20	0·13	0·14
ln development assistance (USD, millions)	0·01	0·02	0·48	0·05	0·03	0·09	0·03	0·02	0·30
Random effects									
Country									
Residual		0·0			0·07			0·06	
Model fit		0·12			0·26			0·33	
AIC	-348·1			214·8			466·5		

Note: Outcome is natural log of the number of publications

Appendix Table 4. Country income group-stratified linear mixed effect models examining the relationship between COVID-19 deaths per 100 population in a country and the number of publications related to COVID-19 and women's well-being including focus on that country six months later.

	Low-income			Lower-middle income			Upper-middle income		
	Estimate	SE	p-value	Estimate	SE	p-value	Estimate	SE	p-value
Within effects									
ln COVID-19 deaths per 100 population	10·01	20·03	0·62	-3·36	3·73	0·37	5·02	1·78	<0·001
Author affiliations with focal country									
Neither lead nor senior	0·55	0·01	<0·001	0·29	0·01	<0·001	0·21	0·01	<0·001
Only lead	0·41	0·05	<0·001	0·34	0·05	<0·001	-0·01	0·06	0·85
Only senior	0·43	0·05	<0·001	0·42	0·07	<0·001	0·30	0·07	<0·001
Both lead and senior/Lead author in single-author paper	0·32	0·02	<0·001	0·13	0·01	<0·001	0·07	0·01	<0·001
Between effects									
ln COVID-19 deaths per 100 population (mean)	15·57	55·86	0·78	5·90	8·23	0·48	-0·85	3·54	0·81
Author affiliations with focal country									
Neither lead nor senior (mean)	0·49	0·07	<0·001	0·33	0·06	<0·001	0·63	0·07	<0·001
Only lead (mean)	1·16	0·46	0·01	0·85	0·23	<0·001	-0·42	0·33	0·22
Only senior (mean)	0·08	0·23	0·73	0·55	0·70	0·44	0·18	0·37	0·64
Both lead and senior/Lead author in single-author paper (mean)	0·15	0·08	0·05	0·10	0·04	0·02	-0·01	0·03	0·80
Gender Inequality Index	-0·25	0·11	0·03	0·14	0·15	0·38	0·11	0·14	0·43
ln development assistance (USD, millions)	0·01	0·02	0·69	0·05	0·03	0·08	0·02	0·03	0·43
Random effects									
Country		0·0			0·07			0·06	
Residual		0·12			0·26			0·33	
Model fit									
AIC	-361·3			200·6			466·4		

Note: Outcome is natural log of the number of publications

Appendix Table 5. Country income group-stratified linear mixed effect models examining the relationship between COVID-19 case fatality ratio in a country and the number of publications related to COVID-19 and women's well-being including focus on that country six months later.

	Low-income			Lower-middle income			Upper-middle income		
	Estimate	SE	p-value	Estimate	SE	p-value	Estimate	SE	p-value
Within effects									
ln COVID-19 case fatality ratio	0·00	0·01	0·73	0·04	0·02	0·09	0·07	0·03	<0·001
Author affiliations with focal country									
Neither lead nor senior	0·54	0·01	<0·001	0·27	0·01	<0·001	0·21	0·01	<0·001
Only lead	0·41	0·05	<0·001	0·32	0·05	<0·001	-0·01	0·06	0·82
Only senior	0·43	0·05	<0·001	0·41	0·07	<0·001	0·32	0·07	<0·001
Both lead and senior/Lead author in single-author paper	0·32	0·02	<0·001	0·13	0·01	<0·001	0·07	0·01	<0·001
Between effects									
ln COVID-19 case fatality ratio (mean)	0·02	0·02	0·31	0·08	0·03	0·30	-0·05	0·20	0·80
Author affiliations with focal country									
Neither lead nor senior (mean)	0·49	0·07	<0·001	0·34	0·06	<0·001	0·69	0·08	<0·001
Only lead (mean)	1·27	0·45	0·01	0·82	0·24	<0·001	-0·42	0·36	0·25
Only senior (mean)	0·14	0·26	0·59	0·64	0·73	0·39	-0·01	0·40	0·99
Both lead and senior/Lead author in single-author paper (mean)	0·14	0·08	0·07	0·09	0·04	0·04	-0·02	0·03	0·47
Gender Inequality Index	-0·34	0·14	0·01	0·07	0·15	0·65	0·12	0·14	0·38
ln development assistance (USD, millions)	-0·01	0·02	0·79	0·04	0·03	0·15	0·01	0·03	0·65
Random effects									
Country		0·0			0·07			0·07	
Residual		0·13			0·27			0·33	
Model fit									
AIC	-285·2			227·8			459·3		

Note: Outcome is natural log of the number of publications
